# The Ly49E Receptor Inhibits the Immune Control of Acute *Trypanosoma cruzi* Infection

**DOI:** 10.3389/fimmu.2016.00472

**Published:** 2016-11-10

**Authors:** Jessica Filtjens, Nicolas Coltel, Sabrina Cencig, Sylvie Taveirne, Els Van Ammel, Aline Van Acker, Tessa Kerre, Patrick Matthys, Tom Taghon, Bart Vandekerckhove, Yves Carlier, Carine Truyens, Georges Leclercq

**Affiliations:** ^1^Laboratory of Experimental Immunology, Ghent University, Ghent, Belgium; ^2^Laboratory of Parasitology, Faculty of Medicine, Université Libre de Bruxelles, Brussels, Belgium; ^3^Laboratory of Immunobiology, Department of Microbiology and Immunology, Rega Institute for Medical Research, KU Leuven – University of Leuven, Leuven, Belgium

**Keywords:** Ly49E receptor, natural killer cells, *Trypanosoma cruzi*, immune control, urokinase plasminogen activator, IFN-γ

## Abstract

The protozoan parasite *Trypanosoma cruzi* circulates in the blood upon infection and invades various cells. Parasites intensively multiply during the acute phase of infection and persist lifelong at low levels in tissues and blood during the chronic phase. Natural killer (NK) and NKT cells play an important role in the immune control of *T. cruzi* infection, mainly by releasing the cytokine IFN-γ that activates the microbicidal action of macrophages and other cells and shapes a protective type 1 immune response. The mechanisms by which immune cells are regulated to produce IFN-γ during *T. cruzi* infection are still incompletely understood. Here, we show that urokinase plasminogen activator (uPA) is induced early upon *T. cruzi* infection and remains elevated until day 20 post-infection. We previously demonstrated that the inhibitory receptor Ly49E, which is expressed, among others, on NK and NKT cells, is triggered by uPA. Therefore, we compared wild type (WT) to Ly49E knockout (KO) mice for their control of experimental *T. cruzi* infection. Our results show that young, i.e., 4- and 6-week-old, Ly49E KO mice control the infection better than WT mice, indicated by a lower parasite load and less cachexia. The beneficial effect of Ly49E depletion is more obvious in 4-week-old male than in female mice and weakens in 8-week-old mice. In young mice, the lower *T. cruzi* parasitemia in Ly49E KO mice is paralleled by higher IFN-γ production compared to their WT controls. Our data indicate that Ly49E receptor expression inhibits the immune control of *T. cruzi* infection. This is the first demonstration that the inhibitory Ly49E receptor can interfere with the immune response to a pathogen *in vivo*.

## Introduction

*Trypanosoma cruzi* is an intracellular protozoan parasite infecting 8–10 million people, especially in endemic areas of Latin America. American trypanosomiasis, also called Chagas disease, mainly results from transmission of parasites from blood-sucking triatomine bugs, leading to a severe chronic disease in 30–40% of infected people. Other transmission ways include transfusion of contaminated blood, organ transplantation, and the congenital route (1, 2). *T. cruzi* infection evolves in two phases: 1) the initial or acute phase, which lasts for approximately 10 weeks after infection and is characterized by a high number of parasites present in blood and tissues, mostly without symptoms though it may be life-threatening, particularly in children, and 2) the chronic phase during which parasites persist lifelong at very low levels in different tissues. This phase is asymptomatic until 30–40% of the individuals develop severe cardiac or digestive damages that may be lethal. The cardiac form is the most frequent manifestation of chronic Chagas disease. It leads to arrhythmia, apical aneurysm, cardiac failure, thromboembolism, and sudden death. Parasite persistence and unbalanced type 1 inflammatory immune response are likely the dominant determinants of Chagas’ cardiomyopathy. Autoantibodies and a hypercoagulability state might also be involved (1–3).

Parasites enter the body as metacyclic trypomastigotes through broken skin, caused by a bug bite or through other cuts and abrasions, or through mucous membranes, including the eyes and mouth. *T. cruzi* trypomastigotes then invade host cells. All types of nucleated cells are potential targets. Inside host cells, they transform into amastigotes, which multiply by binary fission in the cytoplasm. They redifferentiate into trypomastigotes that are released upon cell rupture and disseminate through the bloodstream to infect new cells or are taken up by a triatomine bug (4). To invade the host and the susceptible host cells, the parasite has to pass barriers such as the extracellular matrix thanks to surface or secreted proteases degrading the extracellular matrix (5, 6). The parasite might also indirectly trigger degradation of the extracellular matrix by activating host proteases. One of these is plasmin (6), which is produced from plasminogen by the action of plasminogen activator (7). To do this, the parasite can bind soluble plasminogen to its surface (6, 8). Moreover, production of plasminogen activator by macrophages has been shown to occur during *T. cruzi* infection in mice (9). Besides its role in parasite invasiveness, components of the plasminogen activation system might be involved in chagasic chronic cardiomyopathy. Indeed, patients suffering from chagasic chronic cardiomyopathy present elevated levels of plasminogen and reduced levels of plasminogen activator inhibitor-1 (PAI-1) (10, 11).

Infection of the liver during acute *T. cruzi* infection has been demonstrated both in infected humans and experimentally infected mice. This organ is particularly efficient in controlling parasite multiplication and harbors low numbers of amastigote nests as compared to other organs, although displaying inflammation (12). A major protective cytokine in this process is IFN-γ, as acutely infected IFN-γ knockout (KO) mice present numerous amastigote nests in the liver in contrast to wild-type (WT) mice (12). Liver natural killer (NK) cells have been characterized as rapidly expanding and as early IFN-γ producers. As the infection progresses, conventional T cells and γδ T cells likely provide the IFN-γ necessary for liver protection against *T. cruzi* infection. Furthermore, Duthie and Kahn (13) have shown that both liver NK cells and NKT (including invariant NKT) cells provide protection during *T. cruzi* infection. NKT cells express T cell receptors recognizing glycolipids presented by the monomorphic MHC class-I-like molecule CD1d. When activated, they regulate other immune cells through cell–cell interactions and rapid cytokine production (14). Accordingly, alpha-galactosylceramide-activated NKT cells secrete IFN-γ, which then generally contributes to NK cell activation (15). It has been shown that *T. cruzi* in association with IL-12 activates NKT cells early during mouse infection to produce IFN-γ (16). However, activation of liver NK cells during *T. cruzi* infection and early protection occurs independently of iNKT cells. In contrast, iNKT cells regulate the size of the liver NK cell population, especially during the first days of infection, thereby likely limiting the damaging pro-inflammatory response (17). The protective role of NK cells during *T. cruzi* infection has been further demonstrated in NK-depleted mice, which are characterized by higher parasitemia and shorter survival time (18–22).

Natural killer cells express a repertoire of activating and inhibitory receptors, including Ly49 receptors in mouse. A unique member of this family is the inhibitory Ly49E receptor, which has characteristics that are clearly distinct from other Ly49 family members. Unlike other Ly49 receptors, Ly49E does not bind classical MHC-I ligands (23). Instead, Ly49E is triggered by urokinase plasminogen activator (uPA) (24). Also, Ly49E is the only Ly49 receptor expressed on fetal and neonatal NK cells (25, 26), while in adult tissues, Ly49E expression is largely restricted to NKT cells, skin Vγ3 T cells, and intestinal intraepithelial T lymphocytes (27–29). Conventional peripheral NK cells in adult mice have low Ly49E expression levels (25, 28, 30); however, tissue-resident CD49a^+^CD49b^−^ liver NK cells express high Ly49E levels, and this both in fetal, newborn, and adult mice (31).

Natural killer and NKT cells play an important role in the control of liver parasite burden during the acute phase of *T. cruzi* infection (12, 13). This, combined with the high expression of the inhibitory Ly49E receptor on liver NK and NKT cells (25, 31), with the induction of macrophages to secrete high levels of plasminogen activator upon *T. cruzi* infection (9), and with the triggering of the Ly49E receptor by uPA (24), led us to hypothesize that Ly49E plays a regulatory role in *T. cruzi* infection. We show that plasma uPA levels are increased during *T. cruzi* infection. By comparing WT to Ly49E KO mice, we further demonstrate that young Ly49E KO mice control the infection better than WT mice and this is paralleled by higher IFN-γ production in Ly49E KO mice. Thus, Ly49E expression is harmful in the immune control of *T. cruzi* infection.

## Materials and Methods

### Animals

Heterozygous Ly49E^+/−^ mice (C57BL/6 background) were interbred to obtain homozygous Ly49E^+/+^ (WT) and Ly49E^−/−^ (KO) mice, which were bred separately thereafter (31). Ly49E WT and KO mice were bred in individually ventilated cages in the SPF animal facility of Ghent University (Ghent, Belgium). Mice were sex- and age-matched in each experiment. WT and Ly49E KO mice *T. cruzi* infections were performed at the Université Libre de Bruxelles (ULB; Belgium). Animals were housed in the accredited animal facility, and animal experimentations were performed after approval by and according to the guidelines of the “Université Libre de Bruxelles” Ethic Committee for the use of laboratory animals, adhering to the Belgian legislation on protection of such animals (protocol 529N approved by CEBEA).

Male and female mice at the age of 4–8 weeks were infected with *T. cruzi* blood trypomastigotes and were age matched between WT and Ly49E KO mice. Mice were subcutaneously (s.c.) inoculated by footpad inoculation of 50 (4-week-old mice) or 100 (6- and 8-week-old mice) viable trypomastigotes of the Tulahuen strain (genotype VI).

Parasitemia was determined by microscopic examination of tail vein blood, with a detection limit of 10,000 parasites/ml, as described previously (32). For cytokine and uPA analysis, tail vein blood was withdrawn from non-infected animals (either before infection or from age-matched mice) and at several time points post-infection, as indicated. Blood was collected in heparinized capillaries (Hirschmann, Eberstadt, Germany) and immediately diluted twofold with buffer containing protease inhibitors (NaCl 0.15 M with 13 mM Na citrate, 1 mM tosyl-l-lysyl-chloromethane hydrochloride, and 1000 KIU/ml aprotinin). Subsequently, plasma was obtained upon sample centrifugation and stored at −70°C until use. Survival time and body weight were regularly recorded during infection. Body weight changes were expressed as [(weight on experimental day − weight on day of infection)/weight on day of infection] × 100.

### Cell Preparation and Flow Cytometric Analysis

Spleen and liver lymphocytes were isolated from non-infected and *T. cruzi*-infected WT and Ly49E KO mice that were sacrificed by cervical dislocation. Spleens were disrupted, minced, and passed through a 40-μm cell strainer (Falcon Franklin Lakes, NJ, USA). Erythrocytes were lysed with ammonium–chloride–potassium (ACK) lysing buffer (Invitrogen Corporation, Carlsbad, CA, USA), and cells were washed three times with DPBS. After mechanical disruption of the liver, lymphocytes were isolated using 37.5% Percoll (GE Healthcare, Barrington, IL, USA) density centrifugation. Cells were washed with PBS and counted with trypan blue to exclude dead cells. Cells were flow cytometrically analyzed for cytokine production. Therefore, cells were incubated at 37°C and 5% CO_2_ for 1 h in complete RPMI medium [this refers to RPMI 1640 medium supplemented with 10% FCS, 100 U/ml penicillin, 100 μg/ml streptomycin, 2 mM glutamine, and 50 μM 2-mercaptoethanol (all from Invitrogen Corporation)] supplemented with Brefeldin A (Golgiplug, BD Biosciences). Cells were collected, cell-surface stained, and subsequently permeabilized using Cytofix/Cytoperm reagent (BD Biosciences, San Jose, CA, USA) and intracellularly stained with anti-IFN-γ and anti-TNF-α mAb. Lymphocyte subpopulations were gated as follows: NK cells (CD3^−^NK1.1^+^), NKT cells (CD3^dim^NK1.1^+^), CD8^+^ T cells (CD3^+^NK1.1^−^CD8^+^CD4^−^), CD4^+^ T cells (CD3^+^NK1.1^−^CD8^−^CD4^+^), and γδ^+^ T cells (CD3^+^NK1.1^−^TCRδ^+^). The absolute cell number of IFN-γ- and TNF-α-positive cells in each subpopulation was calculated based on the total viable cell number, the percentage of the representative subpopulation, and the percentage of IFN-γ- or TNF-α-positive cells therein.

Monoclonal antibodies used for labeling were anti-NK1.1 [phycoerythrin (PE)–cyanine-7-conjugated, clone PK136], anti-CD3 (pacific blue-conjugated, clone 145-2C11), anti-γδ-TCR [fluorescein (FITC)-conjugated, clone GL3], anti-CD8α (APC-conjugated, clone Lyt-2), TNF-α (PE-conjugated, clone MPG-XT22), anti-IFN-γ (PE-conjugated, clone XMG1.2) (all from BD Biosciences, San Jose, CA, USA), and anti-CD4 (peridinin chlorophyll protein cyanine dye 5.5-conjugated, clone L3T4, Biolegend). Before staining, the FcR was blocked with anti-FcγRII/III mAb (unconjugated, clone 2.4G2, kindly provided by Dr. J. Unkeless, New York, NY, USA). Live and dead cells were discriminated by the LIVE/DEAD^®^ Fixable Aqua Dead Cell Stain Kit (Invitrogen Corporation). Samples were measured using a BD LSR II flow cytometer and analyzed using FACSDiva 6.1.2 software (BD Biosciences).

### Cytokine Analysis

Plasma cytokine levels were determined using mouse IFN-γ, TNF-α, and IL-12/IL-23p40 Cytometric Bead Array (CBA) flex set kits (BD Biosciences, Belgium), following manufacturer’s instructions. Briefly, capture beads were mixed and added to the unknown samples and serially diluted cytokine standards for 1 h at room temperature. Subsequently, PE-labeled detection antibody was added to the samples, incubated for 1 h at room temperature and washed with wash buffer. Samples were measured on a BD LSR II flow cytometer and analyzed by FCAP Array TM Software (BD Bioscience) to determine the cytokine concentrations of the experimental samples.

### Murine uPA Enzyme-Linked Immunosorbent Assay

The mouse uPA total antigen assay (Innovative research, Novi, MI, USA) was used to determine uPA levels in mouse plasma samples obtained at the indicated day post-infection (dpi). Briefly, samples were added to microtiter plates pre-coated with uPA capture antibody. After 30 min of incubation, plates were washed, and polyclonal anti-murine uPA primary antibody was added. Excess antibody was washed away, and plates were incubated with secondary antibody conjugated to horseradish peroxidase. Finally, 3,3′,5,5′-tetramethylbenzidine substrate was added. Absorbance levels at 450 nm were determined.

### Statistical Analysis

Data were evaluated statistically using Graphpad Prism (Graphpad Software, La Jolla, CA, USA). Datasets were analyzed using the non-parametric two-tailed Mann–Whitney *U*-test. A *p*-value ≤0.05 was considered statistically significant.

## Results

### Urokinase Plasminogen Activator Levels Increase during Acute *T. cruzi* Infection

Nogueira et al. showed that peritoneal macrophages harvested 2 or 3 weeks after *T. cruzi* infection displayed increased plasminogen activator levels, as analyzed by fibrin degradation *in vitro* (9). Fibrinolysis can be induced by both plasminogen activator members, i.e., tissue plasminogen activator (tPA) and uPA. Both tPA and uPA convert plasminogen into plasmin, which in turn degrades fibrin (33). Additionally, uPA can result in proteolysis of extracellular matrix components (33). As we previously showed that uPA triggers the inhibitory Ly49E receptor (24), we first analyzed, in WT male mice, circulating basal uPA levels in mice of different age and whether uPA is induced upon *T. cruzi* infection.

**Figure 1:**
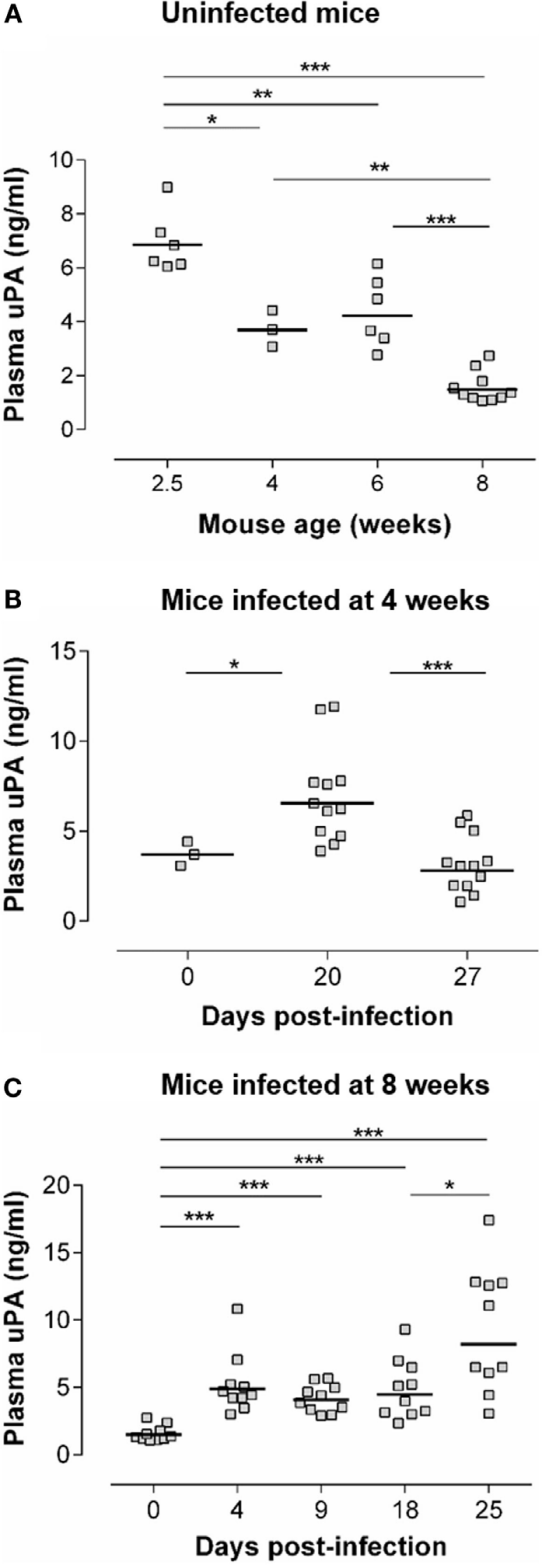
**uPA levels are increased during the acute phase of *T. cruzi* infection**. **(A)** uPA plasma levels were determined by ELISA in WT C57Bl/6 mice of the indicated age. **(B)** Four-week-old WT C57Bl/6 mice were inoculated s.c. with 50 trypomastigotes. uPA plasma levels were determined prior to infection (dpi 0) or at dpi 20 and 27. **(C)** Eight-week-old WT C57Bl/6 mice were inoculated s.c. with 100 trypomastigotes. uPA plasma levels were determined at dpi 0 or at dpi 4, 9, 18, and 25. **(A–C)** Statistically significant differences are indicated with *p ≤ 0.05, **p < 0.01, and ***p < 0.001 (Mann–Whitney *U*-test).

**Figure 2:**
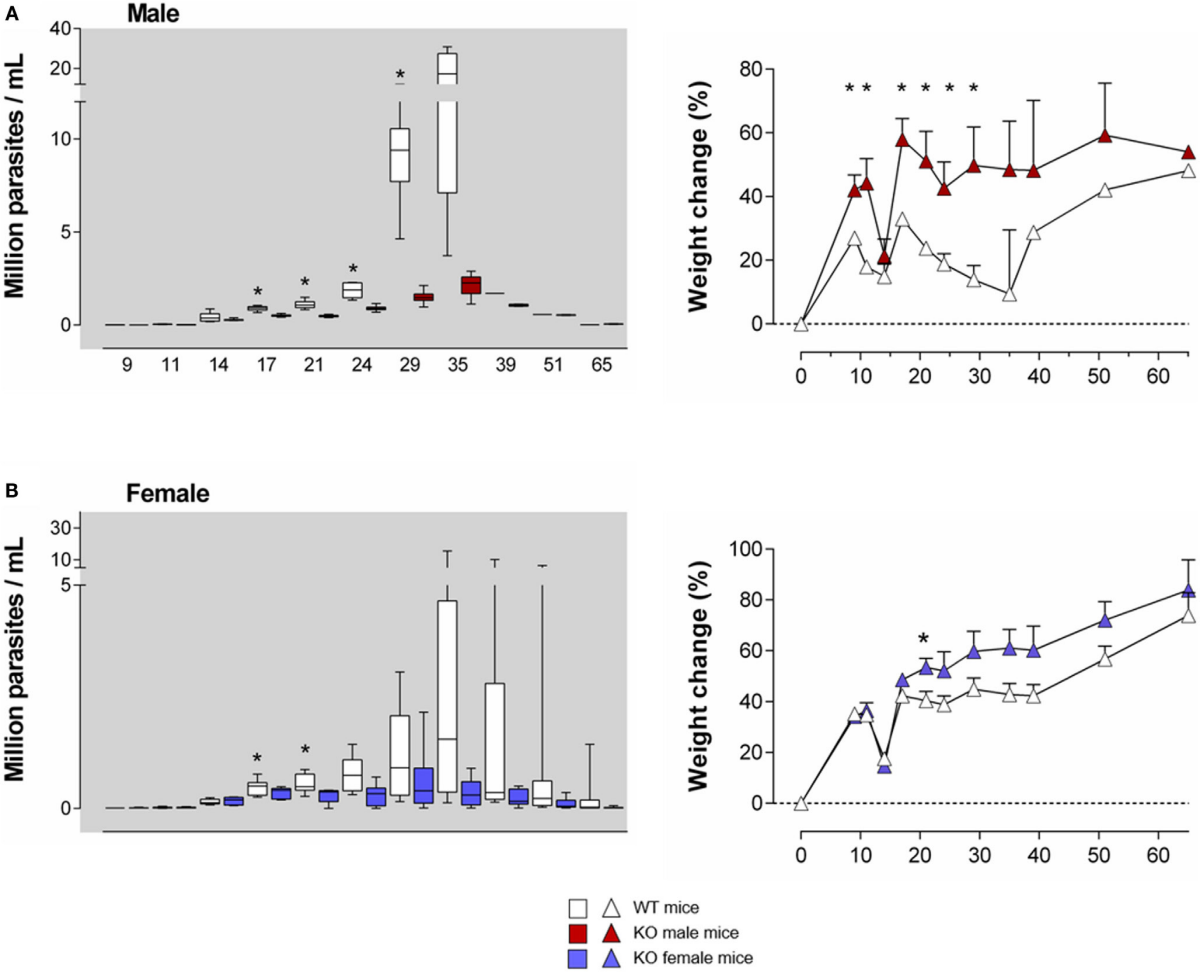
***T. cruzi* infection is better controlled in young Ly49E KO mice compared to their WT controls**. Four-week-old WT and Ly49E KO male **(A)** and female **(B)** mice were inoculated s.c. with 50 trypomastigotes at day 0. Blood parasitemia levels were examined microscopically at the indicated time points, as indicated in the left panels of **(A,B)**. The change in body weight is shown in the right panels (initial weight at the time of infection is set at 0). Results are expressed as Box and Whiskers showing the median from 4 WT and 4 KO male mice and 12 WT and 6 KO female mice. Significant differences between WT and Ly49E KO mice are indicated with *p < 0.05 (Mann–Whitney *U*-test).

**Figure 3:**
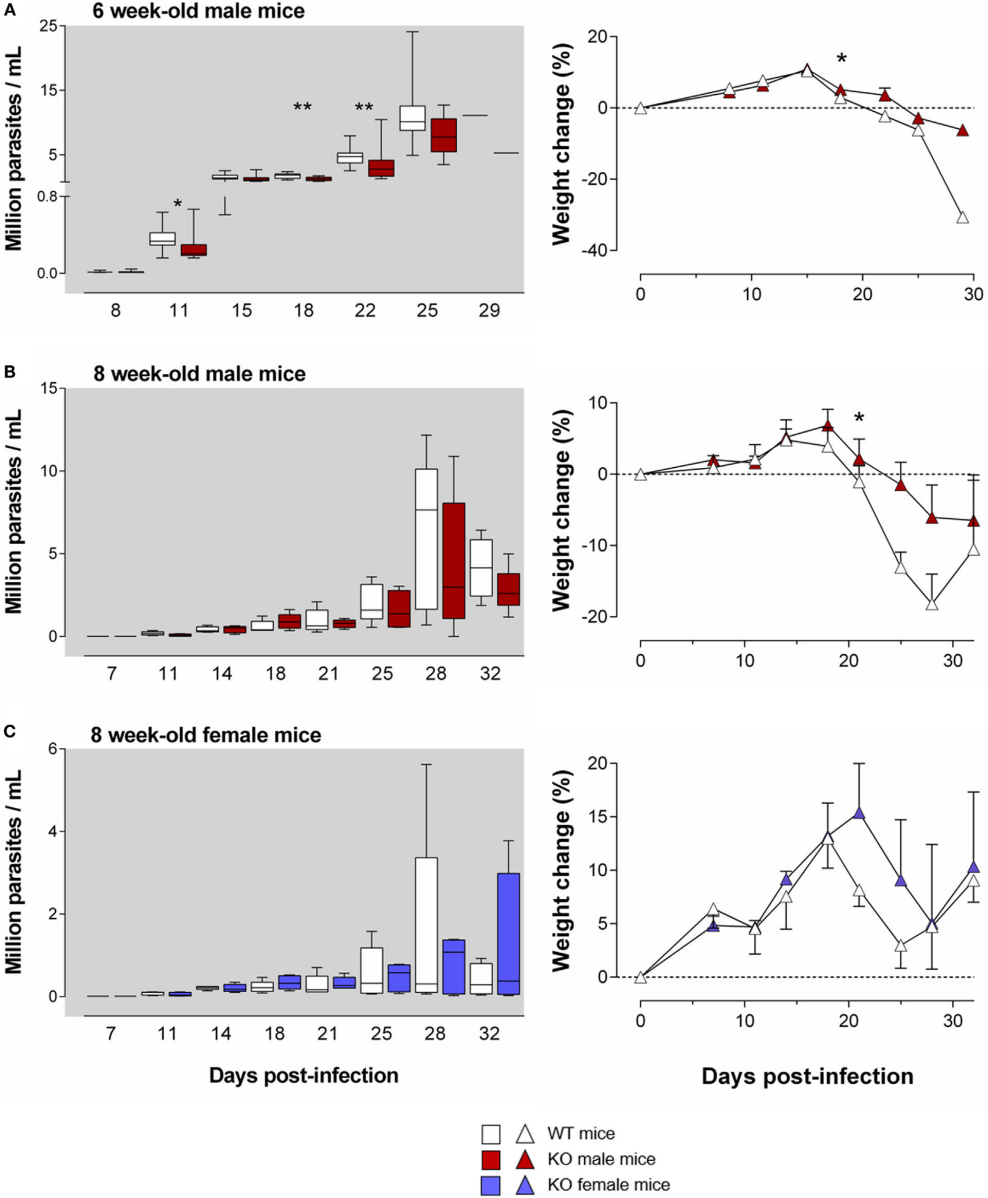
**The harmful effect of the Ly49E receptor on the course of *T. cruzi* infection weakens with mouse age**. WT and Ly49E KO were inoculated s.c. with 100 trypomastigotes at day 0 at the age of 6 **(A)** or 8 weeks **(B,C)**. Blood parasitemia levels were determined at the indicated time points (left panels). Right panels – body weight changes at the indicated days after infection, relative to the initial weight at the time of infection. Results are expressed as Box and Whiskers showing the median from 14 WT and 14 KO 6-week-old mice **(A)**, 5 WT and 5 KO 8-week-old male mice **(B)** and 5 WT and 5 KO 8-week-old female mice **(C)**. Statistically significant differences between WT and Ly49E KO mice are indicated with *p ≤ 0.05 and **p < 0.01 (Mann–Whitney *U*-test).

Figure 1A (upper panel) shows an age-dependent decrease of basal circulating uPA levels in non-infected mice. This has, to the best of our knowledge, not been reported previously. Indeed, the fibrinolytic pathways are overall studied in elderly in relation to risk of thrombosis or in relation to tumor metastasis (34, 35). Nevertheless, it is well-known that the levels of various serum proteins are different in early life as compared with adults, in relation to particular features of their immune system (36). Figures 1B,C show that *T. cruzi* infection was associated with a significant increase of circulating uPA levels. In mice infected at the adult age (Figure 1C), uPA circulating levels increased as soon as dpi 4 and remained elevated at least until dpi 25, when the parasitemia was ascending (cf. Figure 3B for the kinetics of parasitemia). We also observed an elevated plasma uPA level at dpi 20 in mice infected at a younger age (4-week-old – Figure 1B), i.e., also during the ascending phase of parasitemia (cf. Figure 2A). This increase seems less sustained than in adults since uPA returned to basal levels at dpi 27. The reason of this is currently unknown but might relate to different maturity of the immune system with age.

### *T. cruzi* Infection Is Better Controlled in Ly49E KO Mice Compared to Their WT Controls

As Ly49E expression is higher in early life than in adults (26, 28), we first infected 4-week old mice. We used a low *T. cruzi* inoculum size (50 trypomastigotes) to prevent a high mortality rate, since young mice are more susceptible than adults (37). The parasitemia levels were higher in WT male (Figure 2A, left panel) as compared to WT female mice (Figure 2B, left panel), in accordance with the known higher susceptibility of male animals (38). Although no difference in mortality rate or survival time was observed between mouse groups (data not shown), Ly49E KO mice displayed significantly lower parasitemia than WT mice during the acute phase of infection, with the largest difference seen in male mice. The difference of parasite load between WT and Ly49E KO mice was observed rapidly after the parasitemia became patent, reaching 7.5-fold higher mean level in WT as compared to KO mice at dpi 35 [17.3 ± 5.5 × 10^6^ parasites/ml (mean ± SEM), *n* = 4 versus 2.3 ± 0.3 × 10^6^ parasites/ml (mean ± SEM), *n* = 5, *p* = 0.016 (Mann–Whitney *U*-test)]. We also kinetically analyzed the weight of the mice, since cachexia is known to occur during the acute infection (38). As the mice were inoculated with *T. cruzi* at the age of 4 weeks, these mice are still in their growing period. Mice of both WT and KO groups initially gained weight, though somewhat slower in male WT mice (Figures 2A,B, right panels). At the beginning of the patent parasitemic phase (dpi 11–14), WT and KO mice underwent a similar and transient sharp weight loss. When parasitemia became higher (dpi 17 onward) and till the end of the acute phase, Ly49E KO mice displayed significantly lower weight loss than WT mice. The difference was again more pronounced in male mice.

These results indicate that expression of the inhibitory Ly49E receptor is harmful in the control of *T. cruzi* infection of young mice.

As mentioned above, the age of the mice is important for the resultant parasitemia and for the severity of the disease symptoms (37). Moreover, Ly49E expression weakens with age (26, 28). We therefore tested whether the harmful effect of the Ly49E receptor persisted in older mice. We infected 6- and 8-week-old mice in the subsequent experiments, with a slightly higher inoculum size (100 trypomastigotes). As expected, infection of 6- and 8-week-old mice resulted in a trend to lower parasitemia in male WT mice [11 ± 1 × 10^6^ parasites/ml (mean ± SEM), *n* = 12 and 8 ± 2 × 10^6^ parasites/ml (mean ± SEM), *n* = 10, respectively; Figures 3A,B] as compared to 4-week-old male WT mice [17 ± 6 × 10^6^ parasites/ml (mean ± SEM), *n* = 4; cf. Figure 2A], though differences did not reach statistical significance [*p* = 0.127 (6- versus 4-week-old); *p* = 0.057 (8- versus 4-week-old), Mann–Whitney *U*-test]. We still observed in 6-week-old Ly49E KO mice a significantly decreased parasitemia compared to WT mice (Figure 3A). This was associated with a trend in less weight loss of the infected Ly49E KO mice as compared to WT mice, which was significantly different at dpi 18 (Figure 3A). However, in 8-week-old mice, there were no more significant differences in parasitemia between WT and Ly49E KO mice (Figures 3B,C), though we observed a trend of lower parasitemia in Ly49E KO male mice (Figure 3B), as well as a trend for lower weight loss in Ly49E KO mice compared to WT controls in both female and male mice (Figures 3B,C). The mortality rates were similar in WT and Ly49E KO mice, and this in both experiments (8- and 6-week-old mice) (data not shown).

These results show that the harmful effect of Ly49E receptor expression on *T. cruzi* infection weakens with age.

### *T. cruzi*-Infected Ly49E KO Mice Produce Higher Levels of IFN-γ

IFN-γ is the most important cytokine for the control of *T. cruzi* infection (39). Since Ly49E has been shown to inhibit IFN-γ production by cells bearing this receptor (24), we investigated the production of this cytokine, as well as IL-12p40 and TNF-α, in *T. cruzi*-infected Ly49E KO versus WT male mice. We analyzed plasma samples from 4-week-old mice at 20 or 27 days post-infection of *T. cruzi* trypomastigotes or from age-matched non-infected mice. In parallel, parasitemia was analyzed.

**Figure 4:**
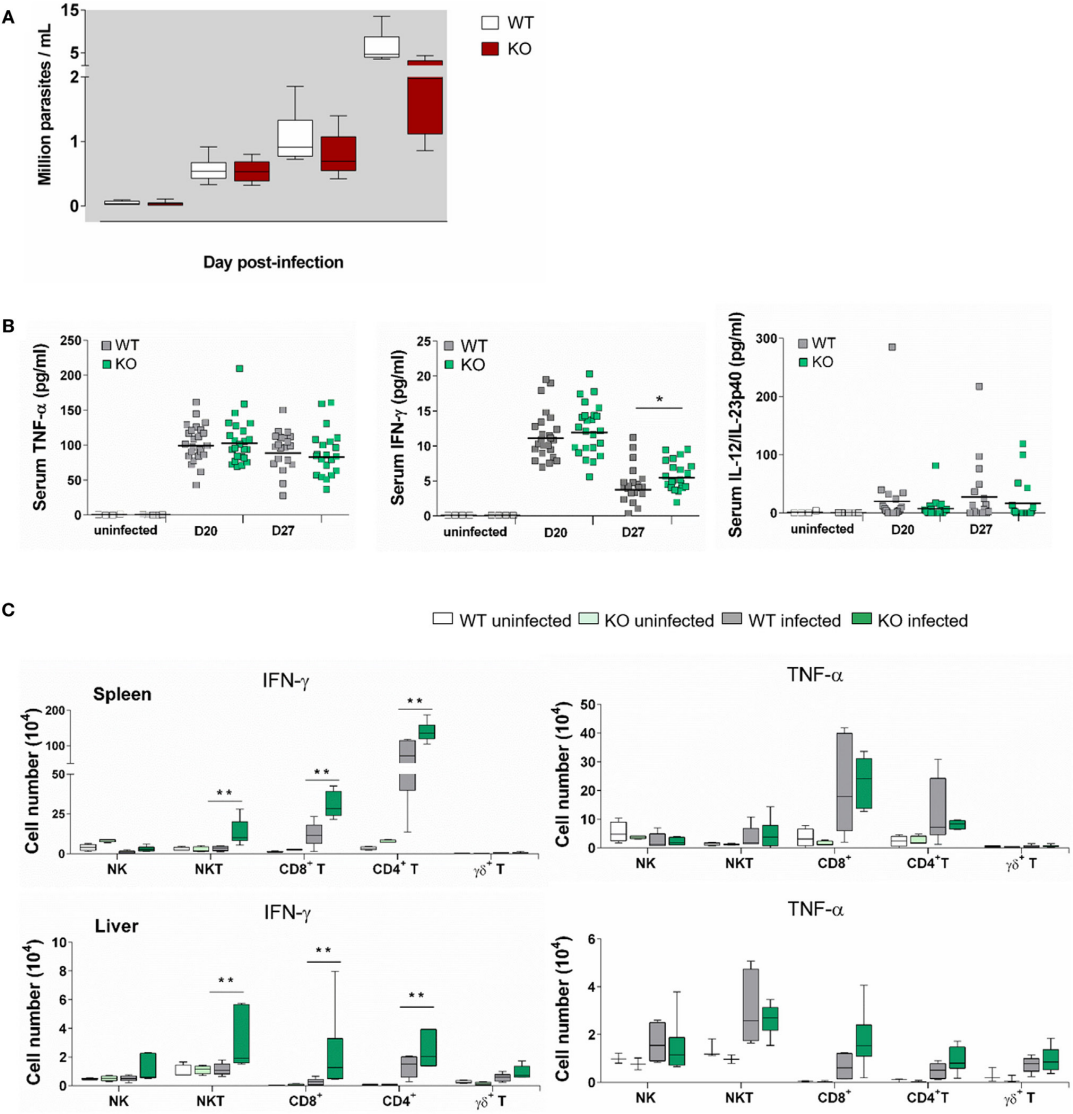
**Enhanced IFN-γ production in *T. cruzi*-infected Ly49E KO mice compared to their WT controls**. Four-week-old male WT and Ly49E KO mice were inoculated s.c. with 50 trypomastigotes at day 0. **(A)** Blood parasitemia levels are shown at the indicated time points (WT, *n* = 24; Ly49E KO, *n* = 24). **(B)** Plasma levels of the indicated cytokines were determined in non-infected mice (WT, *n* = 3; Ly49E KO, *n* = 3) and at day 20 (WT, *n* = 24; Ly49E KO, *n* = 24) and day 27 post-infection (WT, *n* = 21; Ly49E KO, *n* = 21). **(C)** Intracellular IFN-γ and TNF-α were determined by flow cytometry at day 27 post-infection (WT, *n* = 6; Ly49E KO, *n* = 6). The results are presented as the absolute cell number of IFN-γ- (left panels) and TNF-α-positive cells (right panels) in the indicated lymphocyte subpopulations in either spleen (upper panels) or liver (lower panels). Statistical analysis was performed using the Mann–Whitney *U*-test (*p < 0.05 and **p < 0.01).

Both at dpi 21 and dpi 28, parasite blood levels in Ly49E KO were reduced as compared to WT animals (Figure 4A), confirming the harmful effect of the Ly49E receptor on the control of infection (cf. Figure 2A). All tested cytokines were increased in the circulation of infected mice compared to non-infected mice at both examined time points (Figure 4B). TNF-α levels were elevated most (Figure 4B, left panel), but there was no significant difference between WT and Ly49E KO mice. There was also no significant difference for IL-12 p40 in infected WT versus Ly49E KO mice (Figure 4B, right panel). However, for IFN-γ, there was a trend in lower levels in WT compared to Ly49E KO mice at dpi 20, and this became a statistically significant difference at dpi 27 (Figure 4B, middle panel).

We euthanized the mice at dpi 27, i.e., near the peak of parasitemia, and measured *ex vivo* cytokine production in several immune subpopulations of spleen and liver by flow cytometric analysis (Figure 4C). At this time point, CD4^+^ T cells were the main source of IFN-γ in the spleen as well as in the liver of WT-infected mice. CD8^+^ T cells also contributed in the spleen, but not in the liver, while NK and NKT cells did not measurably produce IFN-γ neither in the spleen nor in the liver. By contrast, infected Ly49E KO mice displayed heightened IFN-γ production by CD4^+^ and CD8^+^ T cells but also their NK and NKT cells produced IFN-γ, with NKT cells becoming the main IFN-γ-producing cells in the liver. TNF-α production was also mainly observed in spleen CD4^+^ and CD8^+^ T cells with no difference in infected WT as compared to Ly49E KO mice. The situation is different in the liver where TNF-α was mainly produced by NKT and NK cells, again without difference between mouse groups.

These results show that the protective cytokine IFN-γ is produced less in Ly49E-expressing mice.

## Discussion

The key role of IFN-γ in the control of *T. cruzi* infection has been acknowledged since a long time (39). This cytokine is produced by NKT and NK cells early during infection, followed by CD4^+^ and CD8^+^ T cells when the adaptive immune response develops (12, 13, 18, 20, 21, 40). The protective effect of IFN-γ mainly relies on activation of macrophages and other cells to limit *T. cruzi* replication during the acute phase of infection (40). IFN-γ produced early by NK cells is also important to orchestrate the ongoing adaptive immune response, contributing to differentiation of CD4^+^ Th1 and CD8^+^ T cells required for the control of the parasite multiplication occurring during the acute infection.

Since we have previously shown that the inhibitory Ly49E receptor regulates IFN-γ production (24), we explored the role of this receptor in the control of *T. cruzi* infection by comparing WT to Ly49E KO mice. Contrary to the other inhibitory Ly49 receptors that bind MHC-I molecules (23), Ly49E is triggered by uPA (a plasminogen activator), resulting in inhibition of IFN-γ production (24). Others have shown that plasminogen activator is produced by macrophages upon *T. cruzi* infection (9). We confirmed and extended these results in the present study by showing that plasma uPA levels are increased in *T. cruzi*-infected mice. Of note, uPA levels increased early during the infection, i.e., during the pre-patent and the ascending phases of the parasitemia. This might reflect macrophage activation (9). Contrary to 8-week-old mice, in which plasma uPA levels are still increased at dpi 25, infected 4-week-old mice display basal plasma uPA levels at dpi 27, when parasitemia is high. We can only speculate on the reason for this. First, if uPA initially would promote parasite invasion by triggering plasminogen activation and degradation of extracellular matrix, it has no more sense to maintain increased levels when parasite load is so high and life-threatening for the host. Second, uPA is produced in several cell types, including not only epithelial and endothelial cells but also immune cells. Neutrophils and macrophages are the major uPA-producing immune cell populations. Production of plasminogen activator by macrophages has been shown to occur during *T. cruzi* infection in the mouse (9). uPA-producing cells might become refractory during later stages of *T. cruzi* infection in young mice, resulting in less uPA production when parasitemia is highest. Third, whereas plasma uPA levels are returned to basal levels at later stages of infection in 4-week-old mice, local uPA concentrations in blood vessels of infected tissues might still be increased as also endothelial cells produce uPA during infection. This is of particular interest as CD49b^−^ liver NK cells, the subpopulation of NK cells that mainly expresses Ly49E, are tissue-resident cells that reside in the hepatic sinusoids.

We showed that particularly at young age, i.e., 4–6 weeks after birth, Ly49E KO mice displayed lower *T. cruzi* parasitemia as compared to WT mice. Meanwhile, Ly49E KO mice harbored higher numbers of IFN-γ-positive NKT, CD4 T cells, and CD8 T cells than WT mice near the peak of parasitemia. This was also reflected at the systemic level by a slight increase of circulating IFN-γ levels in Ly49E KO mice at that time point. Since IFN-γ is well-known to be the key cytokine controlling *T. cruzi* infection, such higher IFN-γ production in Ly49E KO is probably at the origin of the lower parasitemia observed in these mice. Our observation that the levels of IL-12, the canonical cytokine inducing IFN-γ, were likely not modified in Ly49E KO mice as compared to WT animals (though levels of bioactive IL-12p70 should be verified), suggests that the higher IFN-γ expression in Ly49E KO mice might be IL-12 independent. This argues for a direct role of the Ly49E receptor on the global IFN-γ response.

We investigated in male Ly49E KO mice infected when they were 4-week old, the liver and splenic immune responses at day 27 post *T. cruzi* infection, i.e., when the inhibition of infection in Ly49E KO mice was more pronounced as compared to WT animals. At this time, Ly49E KO-infected mice harbored a higher proportion of IFN-γ-positive cells than WT-infected animals, with the principal IFN-γ producers being CD4^+^ and CD8^+^ T cells, to a lesser extent NKT cells, whereas NK cells produced hardly IFN-γ. It has a slight systemic impact on circulating IFN-γ levels. These data support that IFN-γ production is inhibited in WT mice expressing the inhibitory Ly49E receptor and not in Ly49E KO mice.

Our current data do not allow to determine if the reduced IFN-γ response of liver and spleen NKT and T cells observed in 4-week-old WT animals at dpi 27 results from a direct inhibition through engagement of Ly49E on these cells at this time point, or indirectly reflects an inhibition that has occurred earlier during infection. Direct inhibition, at this time point, seems less likely since the Ly49E ligand (uPA) has returned to normal levels. Additionally, the fact that the Ly49E receptor is preferentially expressed by NK and NKT cells in younger mice and is also expressed at higher levels on a per cell basis in these mice [(31) and data not shown], also argues for an indirect effect of earlier inhibition of IFN-γ production. Our hypothesis is that the Ly49E receptor is engaged by uPA early during infection, probably as soon as uPA levels are increased, and with a more pronounced effect in young mice harboring a higher proportion of Ly49E^+^ NK cells in the liver than adult mice. What we observe at dpi 27 might be the consequence of earlier Ly49E engagement on NK cells, independently of the present uPA levels that have returned to normal levels. This hypothesis is also supported by the fact that NK cells are rapidly activated during the first days of infection. Indeed, macrophages are among the first cell populations to be infected, resulting in IL-12 release (12). This rapidly activates NK cells to produce IFN-γ, resulting in a Th1 type response of conventional T cells (41–43), necessary for protection against *T. cruzi* infection (12).

Importantly, the beneficial effect of Ly49E deletion was more pronounced in young mice than in adult ones. This likely reflects the variation in Ly49E expression with the mouse age. Indeed, Ly49E is the only Ly49 receptor expressed on neonatal NK cells and is largely absent on conventional peripheral NK cells in adult mice (25, 26), with the exception of CD49b^−^ liver NK cells that express high levels of Ly49E both in newborns and adult mice (31). This latter observation might explain the slight effect that we still observe in 8-week-old mice.

Ly49E KO mice also displayed reduced cachexia during acute *T. cruzi* infection as compared to WT animals. This is in accordance with the relation of weight loss to the parasite burden that we previously showed (38). However, we also showed that TNF-α produced in high amounts during acute *T. cruzi* infection, and not IFN-γ, plays an important role in the cachexia (38). Yet, we did not observe different TNF-α levels between Ly49E KO and WT mice. Nevertheless, the harmful effect of TNF-α on animal weight may rather be regulated by its soluble receptors (44), which we have not measured in this study. Besides, our observations support a differential regulation of IFN-γ and TNF-α production by Ly49E-bearing cells. This is not really surprising since expression of these cytokines is mostly regulated by different transcription factors and post-translational mechanisms (45, 46), but highlights the specific inhibitory action of Ly49E on IFN-γ production.

Our data disclose a modulating role for the inhibitory Ly49E receptor in the control of an infectious disease, wherein Ly49E expression has a deleterious effect for the host regarding its ability to control the *T. cruzi* infection, particularly in young hosts. Keeping this in mind, it is, however, puzzling that Ly49E expressions and plasma uPA levels are higher in early life, a period characterized by increased susceptibility to a lot of infectious diseases, mainly due to the difficulty to mount an optimal type 1 immune response (47). We may hypothesize that the higher expression of the inhibitory Ly49E receptor in early life is necessary to limit damaging inflammation that may occur in response to infections or, particularly in the liver, easily exposed to various microbial products such as endotoxin deriving from the gut (48, 49), and mostly just after birth due to the sudden colonization by microbiota.

## Author Contributions

JF, YC, CT, and GL designed research. JF, NC, SC, ST, EA, and AA performed research and analyzed data. TK, PM, TT, and BV provided reagents. ST, AA, TK, TT, and BV edited the manuscript. JF, CT, and GL wrote the manuscript.

## Conflict of Interest Statement

The authors declare that the research was conducted in the absence of any commercial or financial relationships that could be construed as a potential conflict of interest.

## Abbreviations

dpi, day post-infection; tPA, tissue plasminogen activator; uPA, urokinase plasminogen activator.
